# Persistence of the immune response induced by BCG vaccination

**DOI:** 10.1186/1471-2334-8-9

**Published:** 2008-01-25

**Authors:** Rosemary E Weir, Patricia Gorak-Stolinska, Sian Floyd, Maeve K Lalor, Sally Stenson, Keith Branson, Rose Blitz, Anne Ben-Smith, Paul EM Fine, Hazel M Dockrell

**Affiliations:** 1 London School of Hygiene & Tropical Medicine, Keppel Street, London, WC1E 7HT, UK

## Abstract

**Background:**

Although BCG vaccination is recommended in most countries of the world, little is known of the persistence of BCG-induced immune responses. As novel TB vaccines may be given to boost the immunity induced by neonatal BCG vaccination, evidence concerning the persistence of the BCG vaccine-induced response would help inform decisions about when such boosting would be most effective.

**Methods:**

A randomised control study of UK adolescents was carried out to investigate persistence of BCG immune responses. Adolescents were tested for interferon-gamma (IFN-γ) response to *Mycobacterium tuberculosis *purified protein derivative (M.tb PPD) in a whole blood assay before, 3 months, 12 months (n = 148) and 3 years (n = 19) after receiving teenage BCG vaccination or 14 years after receiving infant BCG vaccination (n = 16).

**Results:**

A gradual reduction in magnitude of response was evident from 3 months to 1 year and from 1 year to 3 years following teenage vaccination, but responses 3 years after vaccination were still on average 6 times higher than before vaccination among vaccinees. Some individuals (11/86; 13%) failed to make a detectable antigen-specific response three months after vaccination, or lost the response after 1 (11/86; 13%) or 3 (3/19; 16%) years. IFN-γ response to Ag85 was measured in a subgroup of adolescents and appeared to be better maintained with no decline from 3 to 12 months. A smaller group of adolescents were tested 14 years after receiving infant BCG vaccination and 13/16 (81%) made a detectable IFN-γ response to M.tb PPD 14 years after infant vaccination as compared to 6/16 (38%) matched unvaccinated controls (p = 0.012); teenagers vaccinated in infancy were 19 times more likely to make an IFN-γ response of > 500 pg/ml than unvaccinated teenagers.

**Conclusion:**

BCG vaccination in infancy and adolescence induces immunological memory to mycobacterial antigens that is still present and measurable for at least 14 years in the majority of vaccinees, although the magnitude of the peripheral blood response wanes from 3 months to 12 months and from 12 months to 3 years post vaccination. The data presented here suggest that because of such waning in the response there may be scope for boosting anti-tuberculous immunity in BCG vaccinated children anytime from 3 months post-vaccination. This supports the prime boost strategies being employed for some new TB vaccines currently under development.

## Background

BCG vaccination is given to young infants in most countries of the world, and the UK has recently changed its vaccination policy to recommend giving BCG primarily to infants in high risk communities [1]. The emphasis upon neonatal vaccination raises the question of the longevity of the protective immune response induced by BCG vaccination. There are few data on the protective effect of infant vaccination on later incidence of pulmonary TB. A review of ten separate studies of vaccine recipients of all ages indicated protection waned after 10 – 15 years [2] but a study in Brazil has shown protection for at least 20 years [3] and a recent analysis of data from North American Indians indicated protection could last for up to 60 years [4]. Though several studies have examined the persistence and waning of BCG-induced tuberculin reactivity [5] there are very few data on the long term persistence of *in vitro *measures of the immune response to BCG.

Recent studies have measured the *in vitro *interferon-gamma (IFN-γ) response to mycobacterial antigens (including M.tb PPD) in diluted whole blood cultures to monitor the immune response to BCG vaccination in adolescents/young adults in the UK and Malawi [6]. UK teenagers, in whom BCG has been shown to provide 50–80% protection against pulmonary TB [7], showed little pre-existing T-cell response to M.tb PPD prior to vaccination, but a clear BCG-attributable increase one year after vaccination. The data presented here is part of a larger data set from an ongoing study comparing the immune responses to BCG vaccination in adolescents and infants in the UK and Malawi, with the purpose of determining correlates of protective efficacy for use in TB vaccine trials. In the UK, BCG vaccination has a protective efficacy of 50–80% [7] while in Malawi it is believed to provide little additional protection to adolescent or adult recipients [8] while there is strong evidence to support the assumption that it is highly efficacious when given to neonates [9]. We have previously determined, in UK and Malawian adolescents, that an increase in BCG attributable M.tb PPD specific IFN-γ response one year post vaccination is associated with the protective efficacy of the BCG vaccine [6]. We now present data to examine more closely the maintenance of the immune response to BCG vaccination among UK teenagers, who were vaccinated either as infants or as adolescents.

## Methods

### Recruitment and study design

This work was carried out within the routine schools BCG programme in Redbridge and Waltham Forest Health Authority (RWFHA) in south-east England, as previously described [10,11]. Approval for this research was given by the RWFHA Local Research Ethics Committee and the Ethics Committee of the London School of Hygiene & Tropical Medicine. Sample collection was carried out between December 2002 and July 2004.

#### Early maintenance of response to BCG vaccination among teenagers

IFN-γ responses in whole blood cultures were measured among 14–15 year olds prior to, and at 3 months and one year after vaccination with Danish strain 1331 BCG. Children were recruited via a letter distributed to parents/guardians, which explained the purpose of the study and contained a written consent form and questionnaire which recorded ethnic group and travel history (time spent outside the UK) [10]. Children with a record of and/or scar evidence of previous BCG vaccination were excluded from the study. All other recruits were given a Heaf skin test using M.tb tuberculin PPD, BP (100,000 U/ml, Evans Medical Limited, Leatherhead, UK), following UK standard procedures [12] at recruitment and 12 months later. Children with a Heaf grade ≥ 2 at recruitment were ineligible for BCG vaccination, and those with a Heaf grade ≥ 3 were referred for active investigation for tuberculosis, following standard procedures [12]. The remaining children were randomly allocated to receive BCG vaccination immediately, or to have it delayed by a year. Participants were recalled for follow-up testing three and twelve months after vaccination. Individuals with Heaf grade < 2, 12 months after recruitment, whose BCG vaccination had been delayed were offered BCG vaccination at the 12 month follow-up.

#### Maintenance of the response 3 years after teenage vaccination

17–18 year olds, vaccinated with Glaxo 1077 BCG (the vaccine in use prior to autumn 2002) at the baseline timepoint of our previous study, and tested by whole blood assay at baseline and one year after vaccination [13], were invited by letter to give a third blood sample, three years after vaccination. Three schools were approached, all of which were participating in the other arms of the study. All participants and their guardians gave their informed consent, and a travel history since the previous blood test was taken.

#### Maintenance of the response 14 years after infant vaccination

IFN-γ responses were measured among 12–14 year olds, 14 years after infant BCG vaccination. Children with a history of BCG vaccination in the first year of life were invited to participate; details of age at vaccination and place or country of vaccination were collected by parental questionnaire and examination of child health records. Unvaccinated children from the main vaccination study acted as controls.

### Whole blood assay and IFN-γ ELISA

Blood collection, whole blood assays and IFN-γ ELISA were carried out as previously described[10]. IFN-γ was measured in 6 day supernatants by ELISA [13]. The limits of detection of the assay were 31 – 2000 pg/ml. Test antigens were *Mycobacteria tuberculosis *(M.tb) PPD (SSI; Batch RT48, lot 191(17–18 year olds study, first and second blood assays) and Batch RT49, lots 204 and 206 (all other assays)) at 5 μg/ml; and for the 14–15 year olds vaccination study, M.tb Antigen 85 A, B, C, purified native antigen 10 μg/ml (Dr J. Belisle, Colorado State University, Fort Collins, USA; through the NIH, NIAID funded programme continued under the HHSN266200400091c "TB Vaccine Testing & Research Materials" contract; lot 01.rv.2.3.21.10Ag85). Controls were PHA (Difco Laboratories/Becton Dickinson, Oxford, UK, 5 μg/ml); streptokinase-streptodornase (SK/SD, Varidase, Wyeth Laboratories, Maidenhead, Berks, UK, 250 U/ml) (17–18 year olds study only); and medium alone (RPMI) as the negative control.

### Statistical analysis

Data were double entered and analysed using STATA 8.1 as previously described [14]. In brief, all values below the limit of detection of the IFN-γ assay < 31 pg/ml were set at 15 pg/ml (the mid point between 0 and 30). Negative control values were subtracted from all results. A positive IFN-γ response was defined as > 62 pg/ml, twice the limit of detection of the assay. The threshold aims to reduce the inclusion of false positive responses and the frequency distribution data from the prevaccination responses among these teenagers indicates that the response threshold is appropriate as previously described [10,13]. To determine if there was a significant vaccine related change in response between the pre and post vaccination time points we compared how many times higher (or lower) the vaccine induced change was than the change in the control group (fold change). Analysis presented here are restricted further to include only individuals who had data at each time point. Ethnic group was categorised as Caucasian, Black, Asian, mixed race, and other. Differences among ethnic groups were further assessed restricting the analysis to children who had not travelled outside the UK.

## Results

### Early maintenance of response to BCG vaccination among teenagers

Two hundred and thirteen teenagers aged 12–14 years were recruited into the study, of whom 189 were eligible for vaccination. Of these, 148 provided a blood sample at baseline and 3 and 12 months after vaccination, and had a Heaf skin test at baseline and 12 months after vaccination. Analysis was restricted to these 148: 78/148 (53%) were male; 123/148 (83%) were Caucasian, the remainder were 'Black' (n = 14), 'Asian' (n = 3), 'mixed race' (n = 2) or 'other' (n = 6). The majority (119/148) had never travelled to tropical areas. Eighty-six (58%) received BCG vaccination at baseline.

Prevalence and magnitude of IFN-γ responses to M.tb PPD and Ag85, and to RPMI and PHA at the different time points are shown in Table 1. Responses to medium alone were generally negative (≤ 8% showing a positive response), and to PHA very high, among both vaccinees and controls, at all three time points.

**Table 1:** Effect of BCG vaccination of UK teenagers on IFN-γ responses to mycobacterial and control antigens. n= 148 (control group n = 62, vaccine group n = 86) except Ag85 where n= 43 (baseline), 50 (3 months), 49 (12 months).

		Time after vaccination					
		
		Baseline		3 months		12 months	
Antigen		Control	Vaccinated	Control	Vaccinated	Control	Vaccinated
M.tb PPD	% responders	23	19	31	87	19	74
	Median pg/ml (IQ range)	0 (0–60)	0 (0–36)	0 (0–103)	488 (150–1178)	0 (0–46)	214 (57–822)
p value Vaccinated versus Control		0.5		< 0.001		< 0.001	
M.tb Ag85	% responders	78	72	90	100	62	96
	Median pg/ml (IQ range)	184 (61–721)	168 (45–426)	813 (221–1340)	1192 (865–2353)	112 (0–262)	808 (386–1865)
p value Vaccinated versus Control		0.67		0.09		0.002	
RPMI	% responders	5	7	8	6	3	6
	Median pg/ml (IQ range)	15 (15)	15 (15)	15 (15)	15 (15)	15 (15)	15 (15)
PHA	% responders	97	100	98	97	87	86
	Median pg/ml (IQ range)	2940 (1432–3692)	3200 (2225–4700)	3807 (1496–4529)	3578 (2610–4092)	2578 (314–3357)	2797 (548–4202)
p value Vaccinated versus Control		0.09		0.76		0.85	

**Figure 1:**
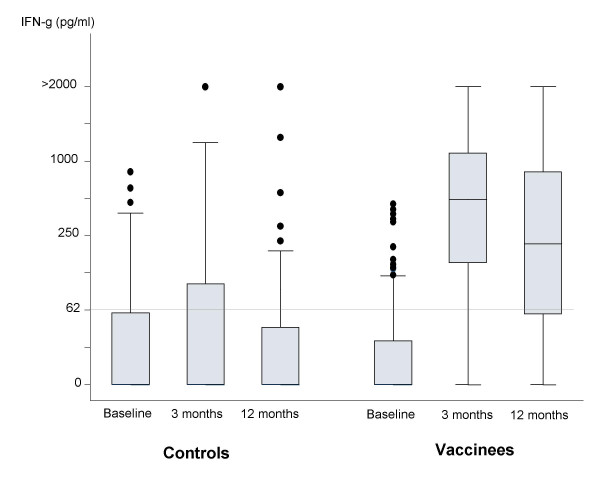
**IFN-γ response to M.tb PPD at baseline, and at 3 and 12 months following BCG vaccination, among teenage unvaccinated controls and vaccinees**. Control subjects n = 62, vaccinated subjects n = 86. Each box shows the median and 25^th ^and 75^th ^centiles. The "whiskers" show the minimum and maximum values other than outliers which are represented as dots above the "whiskers". Grey line represents threshold of positive IFN-γ response (> 62 pg/ml; twice the limit of detection of the assay (31 pg/ml) [13]).

**Figure 2:**
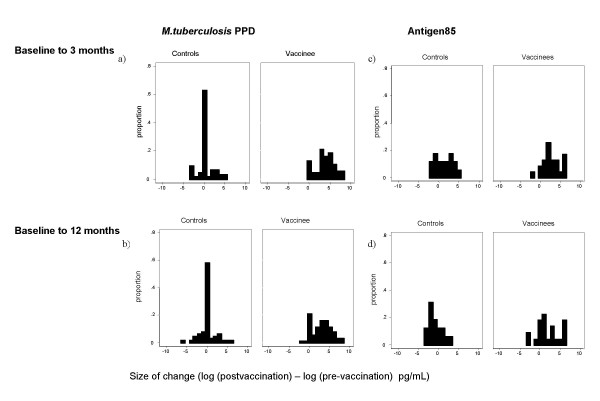
**Change in IFN-γ response to M.tb PPD and M.tb Antigen 85 three and twelve months after BCG vaccination**. Bars represent proportion of the group showing a particular size of change between the timepoints indicated.

Figure 1 shows IFN-γ responses to M.tb PPD at the different time points among controls and vaccinees. Consistent with our previous results [10,13] there were few responders (IFNγ response > 62 pg/ml) to M.tb PPD prior to BCG vaccination, and a marked increase in response at 3 and 12 months after vaccination which is not seen in the control group. Figure 2 shows the frequency distributions of changes in magnitude of IFN-γ response to M.tb PPD between baseline and (a) 3 months and (b) 12 months after BCG vaccination, with control data for comparison. The average increases in vaccinees/controls were 12.4 -fold (p < 0.001)(95% CI 7.7–19.7) and 7.5-fold (p < 0.001)(95% CI 4.5–12.3) at 3 and 12 months respectively. The 12 month response was on average 0.6 times (p = 0.02) (95% CI 0.4–0.9) that of the 3 month response. The size of change in response to M.tb PPD at 12 months was associated with that at 3 months after vaccination (Spearman rank correlation, r = 0.6).

Responses to Ag85 were already highly prevalent among UK teenagers before BCG vaccination (Table 1), which makes the vaccine-induced response less straightforward to interpret. A vaccine-induced effect was still evident, with a mean 2.8-fold increase (p = 0.04)(95% CI 1.0–7.5) in response at 3 months (Figure 2c) and 5.9-fold increase (p = 0.002)(95% CI 2.0–17.8) at 12 months (Figure 2d). The vaccine-induced response to Ag85 was better maintained at 12 months than that to M.tb PPD, with no evidence that the effect of the vaccine waned between 3 and 12 months (p = 0.18). All vaccinated subjects were making a response to Ag85 at three months, and 96% were still responding at 12 months.

An increase in response was also seen among control subjects at 3 months to Ag85 (Figure 2c) (p = 0.02). This was not observed to M.tb PPD (Figure 2a) (p = 0.34). This effect on the response to Ag85 was lost by 12 months (p = 0.27).

### Maintenance of the response 3 years after teenage vaccination

Twenty 17–18 year old teenagers recruited into our earlier study accepted the invitation for further participation. The sample from one subject made no response to test antigens or positive control at the Year 3 test, and was excluded from analysis.

**Figure 3:**
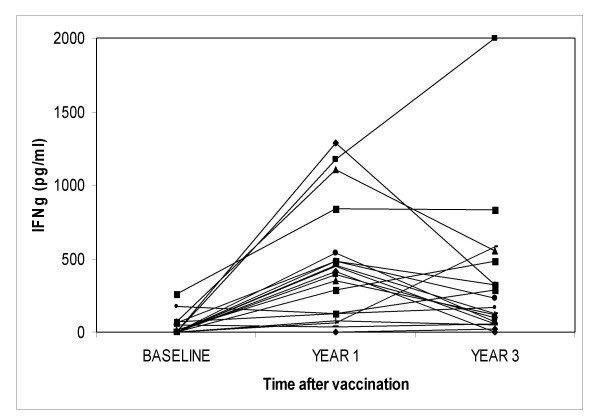
**IFN-γ responses to M.tb PPD in UK teenagers tested before, one and three years after BCG vaccination**. Each triplet of linked points represents IFN-γ response of vaccinated individuals at baseline, one (Year 1) and three (Year 3) years after vaccination with Glaxo 1077 BCG.

Figure 3 shows IFN-γ response to M.tb PPD in the teenagers tested before, 1 and 3 years after, vaccination with Glaxo 1077 BCG. The IFN-γ response to M.tb PPD for the group (n = 19) rose on average 10.2 times (95% CI = 4.9–21.4) from baseline to 1 year. This compares with a mean increase of 9.2 -fold (95% CI 6.8 – 12.5) for the complete group of 424 children vaccinated at the same time in our first study [13]; and of 7.5 -fold (p < 0.001)(95% CI 4.5–12.3) for the children in our second study as reported above. The mean increase from baseline to 3 years was 6.2 times (p < 0.001) (95% CI = 3.3–11.6), consistent with waning of responses between one and three years by a factor of 0.6 (p = 0.10).

IFN-γ responses to a non-mycobacterial antigen, SK/SD, did not increase over the same time period (data not shown); the size of change of IFN-γ response to M.tb PPD was significantly higher (p < 0.001) than that to SK/SD both 1 and 3 years after BCG vaccination, indicating that this is a mycobacterial antigen specific effect.

There was a moderate correlation between the change in response of an individual between baseline and 1 year after vaccination, and between baseline and 3 years after vaccination (Spearman Rank correlation r = 0.58). This change is of the same order as observed 3 and 12 months after vaccination, as reported above. This suggests that the magnitude of the persistent response to vaccination can be quite well predicted by the initial response to vaccination. However, three individuals (16%) who made a response after 1 year made no detectable response after three years. Of 6 individuals (31%) who were non or border line responders to M.tb PPD 1 year after vaccination, 4 were still unresponsive after 3 years, but 2 individuals did make responses. Thus responses to mycobacterial antigens can increase as well as decrease over this period.

### Maintenance of the response 14 years after infant vaccination

**Figure 4:**
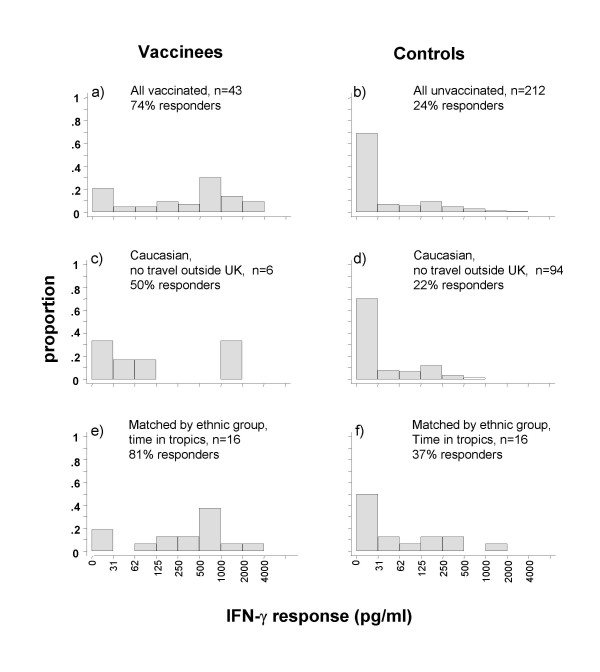
**Frequency distributions of IFN-γ responses to M.tb PPD among 12–14-year olds in the UK, as a function of infant BCG vaccination history, ethnic group and travel history**. Bars represent proportion of each group making an IFN-γ response of particular magnitude.

Forty-three 12–14 year old teenagers with a history of BCG vaccination within the first year of life were recruited. Average time between vaccination and blood test was 14 years, ranging from 12 years 1 month to 14 years 7 months. A group of 212 children of similar age with no history of previous BCG acted as controls. Figure 4 shows IFN-γ responses to M.tb PPD among those who were vaccinated in infancy (Fig 4a) compared to unvaccinated teenagers (Fig 4b). Of those vaccinated as infants, 32/43 (74 %) were responders (> 62 pg/ml), compared to 51/212 (24 %) of those with no history of vaccination (p < 0.001).

Although this suggests that an appreciable proportion of infant vaccinations lead to persistent IFN-γ responses to mycobacterial antigens, it is likely that some of the infant vaccinees may have been vaccinated for reasons (e.g. family history of tuberculosis, immigrant ethnic group) which could provide alternative reasons for positive IFN-γ responses. Twenty four of 43 (56%) were Black or Asian, and our questionnaire revealed that 28/43 (65 %) had visited the tropics, which we have shown to be associated with higher sensitivity to mycobacterial antigens [10]. For 17/43, the BCG vaccination had been administered overseas or could not be confirmed as having been administered in the UK. This group is also likely to have been vaccinated with different strains of BCG.

To clarify the effect of the infant BCG vaccination, the groups were restricted in two ways. Firstly, the infant-vaccinated group was restricted to those who were Caucasian, who had been vaccinated within the first year of life in the UK, and had never travelled to the tropics (n = 6; Figure 4c). These were matched by school to children who were Caucasian and had never travelled to the tropics (n = 94; Figure 4d). Within these groups, 3 (50%) of the vaccinated children and 21 (22%) of the unvaccinated children made an IFN-γ response to M.tb PPD of > 62 pg/ml (p = 0.15, Fisher's exact test); 2 (33%) and 1 (1%) respectively made a response > 500 pg/ml (p = 0.01, Fisher's exact test). Although a statistically significant difference is apparent at the higher level of response, this restriction provided only a very small group of infant-vaccinated children. A further analysis was carried out, matching infant-vaccinated with unvaccinated children by ethnic group, time spent in tropics, geographical area (e.g. South Asia, Caribbean, southern Africa) and where possible exact country visited. Matching also by school was possible for the six infant-vaccinated Caucasian children who had not travelled to tropical countries. This provided 16 matched pairs of vaccinated versus unvaccinated individuals who had travelled to tropical countries (Figures 4e and 4f), together with 6 infant-vaccinated Caucasian students and 94 unvaccinated controls who had not travelled to the tropics. Combining the two groups, and controlling for travel to tropical countries, provided evidence of higher IFN-γ responses among individuals who were infant-vaccinated (odds ratio 5.2 (p = 0.004) [95% CI 1.5–18.3] of having a response > 62 pg/ml, and odds ratio 19.3 (p < 0.001) [95% CI 2.4–155.1], of having a response > 500 pg/ml), compared to individuals who were not.

Some of the Caucasian children who had not travelled to the tropics were also tested with Ag85 (n = 33; unvaccinated n = 27, vaccinated n = 6). There was no difference between the groups in their IFN-γ response (> 62 pg/ml) to Ag85: 74% of the unvaccinated group and 83% of vaccinees made a response. Using a higher response threshold (> 500 pg/ml), 22% of the unvaccinated group and 50% of the vaccinated group made a response. This indicates a higher prevalence of high responses to Ag85 among vaccinees, but low power due to small group sizes did not reveal a statistically significant difference between the two groups.

## Discussion

This study set out to determine the duration of the immune response induced by BCG vaccination in teenagers in the UK, up to 3 years after teenage vaccination or 14 years after infant vaccination. The immune response was measured by IFN-γ response in diluted whole blood culture following 6 days stimulation with M.tb PPD, M.tb Antigen 85, or an appropriate control.

Our results show the effect of infant vaccination in the UK can persist for at least 14 years as measured by the *in vitro *IFN-γ response to M.tb PPD. The magnitude of the measured response, while much higher than that of unvaccinated children, was likely to be lower than the initial response to vaccination, as our study of teenagers showed evidence of highest responses at 3 months after vaccination, with a reduction to 0.6 of the 3 month response by 12 months, and a further reduction to 0.6 of the 12 month response at three years.

In addition to M.tb PPD, we measured responses to Ag85 isolated from *M.tuberculosis*. This antigen has homologues in all known species of mycobacteria and has been identified as a prominent target of the immune response against mycobacteria. It is currently a component of several new tuberculosis vaccines[15]. We have evidence from a previous study that IFN-γ responses to *M. bovis *BCG Ag85 are highly prevalent among UK teenagers before they receive BCG vaccination (manuscript in preparation). A lack of response to M.tb Ag85 in UK cord blood samples, with increasing prevalence of response in blood from BCG-unvaccinated infants over the first year of life (manuscript in preparation), also point to a common environmental exposure to this antigen in the UK. In spite of this, a vaccine-induced increase in response to M.tb Ag85 was observed in our vaccinees, though with a different kinetic of response to that seen to M.tb PPD, the response was maintained at 12 months at its 3 month level. This is likely to follow an initial peak of response in the first month following vaccination, as studies of vaccination of adults from the UK with MVA-85A (and BCG) using *ex vivo *ELISPOT to Ag85A peptides (and PPD) as the measure of response found a marked fall in response after this initial period [15]. In animal model systems it has been demonstrated that prior exposure to different environmental mycobacteria can have an effect on the response to subsequent BCG vaccination either blocking the proliferation of BCG [16] or masking the effect of the BCG by providing as much protection as the vaccine [17]. In the case of Ag85, it may be that repeated environmental exposure to a source of Ag85 maintains response to this antigen and contributes to the persistence of the response to M.tb PPD, of which Ag85 is a major component, so contributing neither to masking nor blocking but to persistence of the response.

Teenagers who had received BCG vaccination in infancy showed no difference in response to Ag85 compared to unvaccinated teenagers at a low threshold of response (< 62 pg/ml), but there was a suggestion that infant BCG vaccination leads to more prevalent high (> 500 pg/ml) responses to Ag85 14 years after vaccination (50% of vaccinated versus 22% unvaccinated), though it should be emphasised this is based upon small numbers and not statistically significant. Larger group sizes would be needed to see if a BCG-induced response to Ag85 persists over many years. Exposure to an environmental species which naturally sensitises individuals to Ag85 makes a persistent vaccine effect more difficult to ascertain with increasing age.

An additional finding from this study was that IFN-γ responses to Ag85 were increased among control subjects at 3 months but not at 12 months. As the same batch of the Ag85 preparation was used at each time point this could reflect some boosting effect by the tuberculin test. Boosting effects have been documented when testing on the day of TST reading or within one month post TST though none using the same assay or at the same time point as in our study [18]. Further human studies of this effect would provide valuable insights.

A recent study has demonstrated that vaccine-induced T-cell response to smallpox antigens can persist for as long as 67 years, in the absence of environmental boosting effects [19]. The authors concluded that this long term memory was attributable mainly to the central memory T-cell population; the effector memory population waned much more rapidly although it could be restored by revaccination. The same study examined the IFN-γ response to PPD, by ELISpot assay, among ten individuals aged 25–63 years, who had received BCG vaccination in childhood, and found it to be maintained both in central and effector memory compartments. The authors conjectured that environmental mycobacterial exposure helped to maintain this response, but no unvaccinated controls were included in the study. Our observed reduction in the magnitude of the peripheral blood IFN-γ response could represent a contraction of the effector memory population with a residual persistent central memory population. We know that at least some individuals in this UK population are exposed to environmental mycobacteria [10,13], which may provide a low-level boosting effect and maintain a small peripheral memory T-cell population to mount the observed IFN-γ response to mycobacterial antigens. Although the unvaccinated control group of teenagers in our study showed some prior sensitivity to M.tb PPD, as expected [10], the children vaccinated in infancy showed a greatly increased IFN-γ response to M.tb PPD as compared to unvaccinated controls. It may be that environmental exposure boosts pre-existing vaccine-induced responses, as well as inducing a response *per se *in unvaccinated individuals.

BCG is the only currently available licensed vaccine against TB, and is typically administered in infancy, including now in the UK. With the worldwide resurgence of TB, it is important to assess longevity of protection afforded by BCG. Information about the induction and maintenance of anti-mycobacterial immune mechanisms by BCG is also relevant for new TB vaccines currently in development [20] as several of these vaccines are designed to act as a booster vaccination following BCG, and such information may inform decisions about the best timing for such boosting.

## Conclusion

In conclusion, BCG vaccination of infants in the UK provokes a response in the majority of vaccinees which can persist for at least 14 years, as measured by the IFN-γ response to mycobacterial antigens of peripheral blood cultures. The maintenance of memory T-cell IFN-γ responses to Ag85 implies that BCG vaccination primes T-cell responses that could be boosted by the new vaccines incorporating Ag85A or Ag85B that are currently under development or entering trials. However, recent studies in Malawi show that, against an already high response to mycobacterial antigens, BCG vaccination induces an increased IFN-γ response to mycobacterial antigens that is detectable at 3 months, but is much lower by 12 months [21]. This may reflect a reduced ability of the Malawian group to generate new longer term central memory cells due to high environmental mycobacterial exposure over-stimulating an activated effector cell population. As the majority of new cases of TB are in the tropics, and this is where BCG has consistently failed to provide protection against adult disease, it is important to better understand these factors to arrive at a new vaccine which will succeed where BCG has failed.

## List of abbreviations

Ag85: Antigen 85

BCG: Bacille Calmette-Guérin

IFN-γ : Interferon gamma

M.tb PPD: *Mycobacterium tuberculosis *purified protein derivative

PHA: phytohaemaglutinin

SK/SD: streptokinase/streptodornase

TB: tuberculosis

## Competing interests

The author(s) declare that they have no competing interests.

## Authors' contributions

The study was designed by HD and PF with help from PG-S, RW, AB-S and SF. Protocols were designed by PG-S, RW and AB-S. Statistical analysis was carried out by SF. Computer programmes were designed by KB. Co-ordination of the school study was carried out by RW, PG-S, RB, BN, SL, MH and CS. Immunological testing was carried out by ML, SS, PG-S and RW. Data interpretation and writing of the manuscript was carried out by PG-S, RW, SF, AB-S, PF and HD. All authors have read and approved the final manuscript.

## Pre-publication history

The pre-publication history for this paper can be accessed here:


